# Crystal structure of (1,3-thia­zole-2-carboxyl­ato-κ*N*)(1,3-thia­zole-2-carb­oxy­lic acid-κ*N*)silver(I)

**DOI:** 10.1107/S2056989019000124

**Published:** 2019-01-11

**Authors:** Natthaya Meundaeng, Apinpus Rujiwatra, Timothy J. Prior

**Affiliations:** a Department of Chemistry, Faculty of Science, King Mongkut’s Institute of Technology Ladkrabang, Bangkok, 10520, Thailand; b Department of Chemistry, Faculty of Science, Chiang Mai University, Chiang Mai, 50200, Thailand; c Chemistry, University of Hull, Kingston upon Hull, HU6 7RX, UK

**Keywords:** silver, 2-thia­zole­carb­oxy­lic acid, hydrogen bonding, crystal structure

## Abstract

The linear two-coordinate silver (I) complex [Ag(C_4_H_2_NO_2_S)(C_4_H_3_NO_2_S)] or [Ag(2-Htza)(2-tza)] is reported (2-Htza = 1,3-thia­zole-2-carb­oxy­lic acid). The Ag^I^ ion is coordinated by two heterocyclic N atoms from two ligands in a linear configuration, forming a discrete coordination complex. There is an O—H⋯O hydrogen bond between 2-tza^−^ and 2tzaH of adjacent complexes. The hydrogen atom is shared between the two oxygen atoms. This inter­action produces a hydrogen-bonded tape parallel to the [110] direction, which is augmented through inter­molecular C—H⋯O hydrogen-bonding inter­actions between the bound thia­zole groups. There is a further rather long Ag⋯O inter­action [2.8401 (13) Å, compared with a mean of 2.54 (11) Å for 23 structures in the CSD] that assembles these tapes into columns, between which there are C—H⋯π inter­actions, leading to the formation of a three-dimensional supra­molecular architecture.

## Abstract 2

The Ag^I^ ion is coordinated by two heterocyclic N atoms from two ligands in a linear configuration, forming a discrete coordination complex. There is an O—H⋯O hydrogen bond between 2-tza^−^ and 2tzaH of adjacent complexes. The hydrogen atom is shared between the two oxygen atoms.

## Chemical context   

1,3-Thia­zoles have been known for over a century and many of their derivatives exhibit potential applications, particularly in drug design and biological activity (Ayati *et al.*, 2015 ▸; Kashyap *et al.*, 2012 ▸). The thia­zole­carb­oxy­lic acids have also received attention as ligands in complexes of the first-row transition metals. This is due to the co-presence of the heterocyclic ring and the carboxyl group providing various coordination modes (Frija *et al.*, 2016 ▸). They also favour the assembly of supra­molecular architectures by establishing a variety of non-covalent inter­actions *e.g.* hydrogen bonding and π–π stacking inter­actions (Desiraju, 2002 ▸; Sherrington & Taskinen, 2001 ▸; Blake *et al.*, 1999 ▸). Recently, we reported the syntheses and structural features of Co^II^, Ni^II^, and Cu^II^ complexes with thia­zole-4-carboxyl­ate (Meundaeng *et al.*, 2016 ▸) and thia­zole-5-carboxyl­ate (Meundaeng *et al.*, 2017 ▸). Herein we report the synthesis and crystal structure of the Ag^I^ complex with thia­zole-2-carb­oxy­lic acid (2-tza).
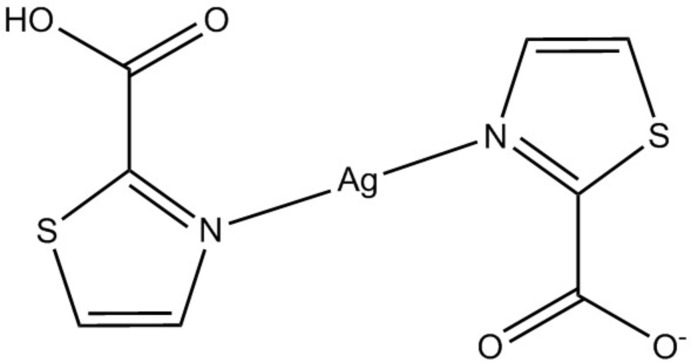


## Structural commentary   

**Figure 1:**
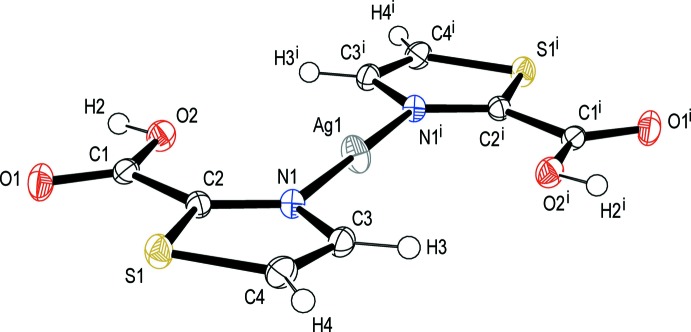
Mol­ecular structure of [Ag(2-Htza)(2-tza)] with 50% probability ellipsoids showing the atom-labelling scheme. Symmetry code: (i) –x, 1 − *y*, 1 − *z*.

The monomeric complex of the title compound crystallizes in the monoclinic space group *P2_1_/c.* The asymmetric unit contains one Ag^I^ ion and one 2-tza ligand which is formally 2-tza(H)_1/2_. The whole mol­ecular structure can be generated by an inversion centre; the Ag^I^ atom is located at the 2*a* Wyckoff position (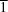) (Fig. 1 ▸). The Ag^I^ centre shows a linear coordin­ation with two 2-tza ligands coordinating through the heterocyclic N atoms with an Ag—N bond length of 2.1463 (14) Å. Statistically one of these ligands has an appended carb­oxy­lic acid and the other a carboxyl­ate. A rather long Ag⋯O2 inter­action is also observed with the distance of 2.8401 (13) Å. This is significantly larger than the mean value [2.54 (11) Å] of the Ag⋯O=C distances in the Cambridge Database (version 5.37 up to October 2018; Groom *et al.*, 2016 ▸; 23 hits, silver bound by two nitro­gen atoms, Ag⋯O=C distance recorded) and suggests that the inter­action between the carbonyl and the silver atom is very weak. No inter­actions between the Ag centres are observed.

## Supra­molecular features   

**Figure 2:**
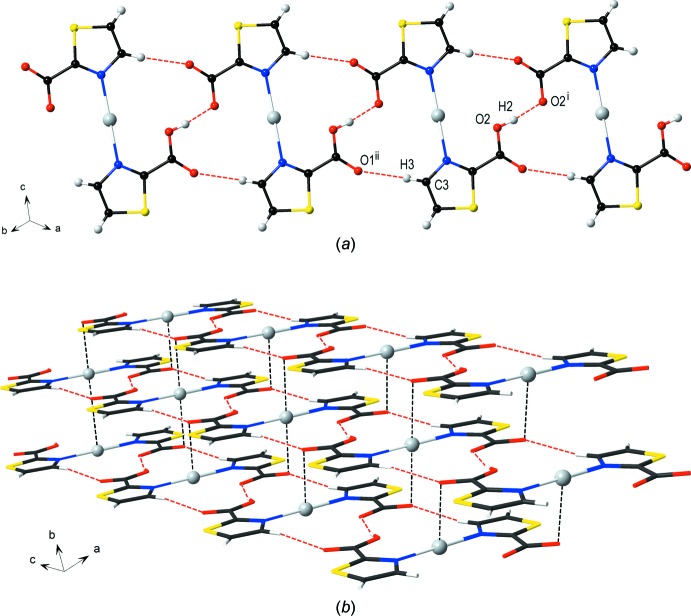
View of (*a*) hydrogen-bonding inter­actions (red dashed lines) leading to the formation of a one-dimensional tape along the [110] direction [symmetry codes: (i) −*x* + 1, −*y*, −*z* + 1, (ii) *x* − 1, *y* + 1, *z*] and (*b*) the weak Ag⋯O inter­actions (black dashed lines) holding each tape into a column.

**Figure 3:**
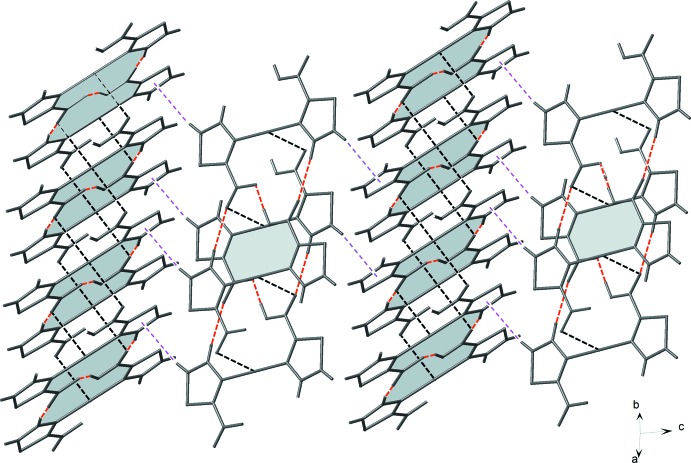
View of supra­molecular inter­actions; hydrogen bonds between the discrete units (red dashed lines), Ag⋯O inter­actions between adjacent one-dimensional tapes (black dashed lines) and C—H⋯π inter­actions between adjacent columns (purple dashed lines).

In order to balance charge in this structure, the 2-tza ligand must be half protonated but we see no evidence for crystallographic ordering of the hydrogen position. In late stages of refinement, a maximum of electron density in the difference-Fourier map was present located 0.897 Å from the atom O2. This was modelled as a half occupied hydrogen atom. Thus, the overall composition is Ag^+^(2-Htza)(2-tza^−^). The carboxyl­ate is located close to a second symmetry-equivalent carboxyl­ate generated by the symmetry operation 1 − *x*, −*y*, 1 − *z*. Statistically, one of these two groups is protonated. The close approach facilitates the formation of a linear hydrogen bond between them (Table 1 ▸) and the O⋯O distance of 2.470 (3) Å strongly suggests that there is a hydrogen bond. Strangely, it is not the case that the O2—H2 distance is half the O2⋯O2^i^ distance as is sometimes observed in similar systems (Leiva *et al.*, 1999 ▸; Deloume *et al.*, 1977 ▸). The partially occupied H2 atom is clearly identified from a Fourier map at the closer distance to the atom O2. Furthermore, the carboxyl O1 atom serves as a hydrogen-bonding acceptor with the heterocyclic H3 atom (C*sp* ^2^—H⋯C=O) of an adjacent discrete mol­ecule (Table 1 ▸). The occurrence of these hydrogen-bonding inter­actions results in the formation of a one-dimensional tape along the [110] direction (Fig. 2 ▸ *a*). This tape is inclined at an angle of 55.278 (19)° with the (001) plane. Each tape is connected through an Ag⋯O1 inter­action [*d*(Ag⋯O1) = 2.9606 (14) Å], generating columns of complex mol­ecules along the [100] direction (Fig. 2 ▸ *b*). These columns are further linked *via* C—H⋯π inter­actions between the thia­zole rings (Table 1 ▸), leading to the formation of a three-dimensional supra­molecular architecture (Fig. 3 ▸).

## Database survey   

A search of the Cambridge Structural Database (CSD version 5.37 up to October 2018; Groom *et al.*, 2016 ▸) revealed a rather small number of previous reports of metal-containing compounds with thia­zole­carb­oxy­lic acids: (i) there are three reports of thia­zole-2-carb­oxy­lic acid (2-tza) structures, *i.e.* two tin^IV^ complexes (Yin & Wang, 2004 ▸; Yin *et al.*, 2005 ▸) and a Zn^II^ complex (Rossin *et al.*, 2011 ▸); (ii) three reports of structures with thia­zole-4-carb­oxy­lic acid (4-tza) *i.e.* Cu^II^ and Zn^II^ complexes (Rossin *et al.*, 2011 ▸) and Co^II^, Ni^II^, Cu^II^ complexes (Meundaeng *et al.*, 2016 ▸) and one with Sn^IV^ (Gao *et al.*, 2016 ▸); and two reports of thia­zole-5-carb­oxy­lic acid (5-tza) complexes each with Cu^II^ (Rossin *et al.*, 2014 ▸; Meundaeng *et al.*, 2017 ▸). While the 4-tza ligand provides a predictable [*N*,*O*]-chelating mode of coordination and the 5-tza ligand exhibits bridging ability through its aromatic N atom and carboxyl O atom, the coordination of the 2-tza ligand to the metal occurs through *O*-monodentate, [*N*,*O*]- and [*O*,*O*]-chelating modes. So far, the S atom on the thia­zole ring has been entirely innocent in the chemistry described. Compared to those first-row transition metals, the softer Ag^I^ ion could be a good candidate for the exploration of the coordination chemistry of these ligands, particularly the 2-tza ligand, as it can possibly bind to the metal ion in various modes of coordination: *N*- and *O*-monodentate, [*N*,*O*]-, [*O*,*O*]- and [*S*,*O*]-chelating modes.

## Synthesis and crystallization   

AgNO_3_ (0.0170 g, 0.100 mmol) and 2-Htza (0.0129 g, 0.100 mmol) were dissolved in 5.0 mL of deionized water in a small vial (*ca* 16 mm in diameter). The vial was left undisturbed at ambient temperature for three days during which colourless block-shaped crystals of the title compound crystallized and were isolated for X-ray data collection.

## Refinement   

Crystal data, data collection and structure refinement details are summarized in Table 2 ▸. The hy­droxy H atom was positioned geometrically (O—H = 0.84) and refined as riding, with *U*_iso_(H) = 1.5*U*_eq_(O). The C-bound H atoms were refined isotropically, with *U*_iso_(H) = 1.2*U*_eq_(C).

## Supplementary Material

Crystal structure: contains datablock(s) I. DOI: 10.1107/S2056989019000124/nk2248sup1.cif

Structure factors: contains datablock(s) I. DOI: 10.1107/S2056989019000124/nk2248Isup2.hkl

Supporting information file. DOI: 10.1107/S2056989019000124/nk2248Isup3.mol

CCDC reference: 1888609

Additional supporting information:  crystallographic information; 3D view; checkCIF report

## Figures and Tables

**Table 1:** Hydrogen-bond geometry (Å, °) *Cg* is the centroid of the S1/N1/C2–C4 ring.

*D*—H⋯*A*	*D*—H	H⋯*A*	*D*⋯*A*	*D*—H⋯*A*
O2—H2⋯O2^i^	0.84	1.65	2.470 (3)	165
C3—H3⋯O1^ii^	0.92	2.37	3.280 (2)	170
C4—H4⋯*Cg* ^iii^	0.91 (3)	2.90 (2)	3.688 (2)	146 (1)

Symmetry codes: (i) 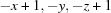; (ii) 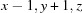; (iii) 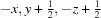.

**Table 2:** Experimental details

Crystal data	
Chemical formula	[Ag(C_4_H_2_NO_2_S)(C_4_H_3_NO_2_S)]
*M*_r_	365.13
Crystal system, space group	Monoclinic, *P*2_1_/*c*
Temperature (K)	150
*a*, *b*, *c* (Å)	5.8613 (9), 5.0180 (6), 18.278 (3)
β (°)	98.303 (13)
*V* (Å^3^)	531.94 (14)
*Z*	2
Radiation type	Mo *K*α
μ (mm^−1^)	2.29
Crystal size (mm)	0.25 × 0.18 × 0.10

Data collection	
Diffractometer	Stoe IPDS2
Absorption correction	Multi-scan (*SORTAV*; Blessing, 1995 ▸)
*T*_min_, *T*_max_	0.868, 0.927
No. of measured, independent and observed [*I* > 2σ(*I*)] reflections	3482, 1691, 1354
*R*_int_	0.026
(sin θ/λ)_max_ (Å^−1^)	0.725

Refinement	
*R*[*F* ^2^ > 2σ(*F* ^2^)], *wR*(*F* ^2^), *S*	0.020, 0.044, 0.90
No. of reflections	1691
No. of parameters	82
H-atom treatment	H atoms treated by a mixture of independent and constrained refinement
Δρ_max_, Δρ_min_ (e Å^−3^)	0.45, −0.34

Computer programs: *X-AREA* (Stoe & Cie, 2002 ▸), *SORTAV* (Blessing, 1995 ▸), *SHELXT* (Sheldrick, 2015*a* ▸), *SHELXL2014/7* (Sheldrick, 2015*b* ▸), *ORTEP-3 for Windows* and *WinGX* (Farrugia, 2012 ▸) and *CrystalMaker* (Palmer, 2014 ▸).

## supplementary crystallographic information

### Crystal data

**Table:**

[Ag(C_4_H_2_NO_2_S)(C_4_H_3_NO_2_S)]	*F*(000) = 356
*M**_r_* = 365.13	*D*_x_ = 2.280 Mg m^−^^3^
Monoclinic, *P*2_1_/*c*	Mo *K*α radiation, λ = 0.71073 Å
*a* = 5.8613 (9) Å	Cell parameters from 3235 reflections
*b* = 5.0180 (6) Å	θ = 3.5–34.2°
*c* = 18.278 (3) Å	µ = 2.29 mm^−^^1^
β = 98.303 (13)°	*T* = 150 K
*V* = 531.94 (14) Å^3^	Block, colourless
*Z* = 2	0.25 × 0.18 × 0.10 mm

### Data collection

**Table:**

Stoe IPDS2 diffractometer	1354 reflections with *I* > 2σ(*I*)
Detector resolution: 6.67 pixels mm^-1^	*R*_int_ = 0.026
ω–scans	θ_max_ = 31.0°, θ_min_ = 3.5°
Absorption correction: multi-scan (SORTAV; Blessing, 1995)	*h* = −8→8
*T*_min_ = 0.868, *T*_max_ = 0.927	*k* = −7→6
3482 measured reflections	*l* = −21→26
1691 independent reflections	

### Refinement

**Table:**

Refinement on *F*^2^	0 restraints
Least-squares matrix: full	Hydrogen site location: inferred from neighbouring sites
*R*[*F*^2^ > 2σ(*F*^2^)] = 0.020	H atoms treated by a mixture of independent and constrained refinement
*wR*(*F*^2^) = 0.044	*w* = 1/[σ^2^(*F*_o_^2^) + (0.0208*P*)^2^] where *P* = (*F*_o_^2^ + 2*F*_c_^2^)/3
*S* = 0.90	(Δ/σ)_max_ < 0.001
1691 reflections	Δρ_max_ = 0.45 e Å^−^^3^
82 parameters	Δρ_min_ = −0.33 e Å^−^^3^

### Special details

**Table:**

Geometry. All esds (except the esd in the dihedral angle between two l.s. planes) are estimated using the full covariance matrix. The cell esds are taken into account individually in the estimation of esds in distances, angles and torsion angles; correlations between esds in cell parameters are only used when they are defined by crystal symmetry. An approximate (isotropic) treatment of cell esds is used for estimating esds involving l.s. planes.

### Fractional atomic coordinates and isotropic or equivalent isotropic displacement parameters (Å^2^)

**Table:**

	*x*	*y*	*z*	*U*_iso_*/*U*_eq_	Occ. (<1)
Ag1	0.0000	0.5000	0.5000	0.02294 (6)	
S1	0.39928 (7)	0.66825 (9)	0.30440 (3)	0.01892 (9)	
O2	0.3909 (2)	0.1980 (3)	0.47470 (7)	0.0195 (3)	
H2	0.4842	0.0819	0.4938	0.029*	0.5
O1	0.6413 (2)	0.2412 (3)	0.39263 (8)	0.0245 (3)	
N1	0.1394 (2)	0.6325 (3)	0.40394 (8)	0.0153 (3)	
C4	0.1715 (3)	0.8824 (4)	0.30037 (11)	0.0192 (3)	
H4	0.1351 (11)	1.009 (4)	0.2649 (10)	0.023*	
C3	0.0518 (3)	0.8370 (4)	0.35772 (10)	0.0169 (3)	
H3	−0.076 (3)	0.934 (2)	0.3650 (2)	0.020*	
C2	0.3237 (2)	0.5272 (3)	0.38216 (9)	0.0140 (3)	
C1	0.4673 (3)	0.3028 (3)	0.41895 (10)	0.0160 (3)	

### Atomic displacement parameters (Å^2^)

**Table:**

	*U*^11^	*U*^22^	*U*^33^	*U*^12^	*U*^13^	*U*^23^
Ag1	0.01955 (8)	0.03224 (11)	0.01899 (10)	0.00117 (9)	0.00935 (6)	0.00427 (10)
S1	0.01923 (17)	0.0192 (2)	0.0203 (2)	0.00206 (15)	0.00930 (15)	0.00304 (17)
O2	0.0197 (5)	0.0164 (6)	0.0221 (7)	0.0033 (4)	0.0023 (5)	0.0040 (5)
O1	0.0193 (5)	0.0224 (6)	0.0331 (8)	0.0073 (5)	0.0078 (5)	0.0006 (6)
N1	0.0140 (5)	0.0158 (7)	0.0162 (7)	0.0018 (5)	0.0028 (5)	−0.0001 (6)
C4	0.0205 (7)	0.0149 (8)	0.0216 (9)	0.0025 (6)	0.0015 (7)	0.0040 (7)
C3	0.0161 (6)	0.0153 (7)	0.0189 (8)	0.0028 (6)	0.0012 (6)	−0.0005 (7)
C2	0.0133 (6)	0.0137 (7)	0.0152 (7)	0.0005 (5)	0.0031 (5)	0.0007 (7)
C1	0.0149 (6)	0.0128 (7)	0.0195 (8)	0.0011 (5)	0.0000 (6)	−0.0023 (7)

### Geometric parameters (Å, º)

**Table:**

Ag1—N1^i^	2.1463 (14)	N1—C2	1.3151 (19)
Ag1—N1	2.1463 (14)	N1—C3	1.380 (2)
S1—C2	1.7026 (17)	C4—C3	1.362 (2)
S1—C4	1.7071 (18)	C4—H4	0.91 (3)
O2—C1	1.283 (2)	C3—H3	0.92 (2)
O2—H2	0.8400	C2—C1	1.505 (2)
O1—C1	1.2280 (19)		
			
N1^i^—Ag1—N1	180.00 (8)	C4—C3—N1	114.09 (14)
C2—S1—C4	90.10 (8)	C4—C3—H3	123.0
C1—O2—H2	109.5	N1—C3—H3	123.0
C2—N1—C3	111.27 (14)	N1—C2—C1	126.61 (14)
C2—N1—Ag1	123.33 (12)	N1—C2—S1	114.18 (13)
C3—N1—Ag1	125.39 (10)	C1—C2—S1	119.21 (11)
C3—C4—S1	110.35 (14)	O1—C1—O2	127.78 (16)
C3—C4—H4	124.8	O1—C1—C2	117.10 (15)
S1—C4—H4	124.8	O2—C1—C2	115.12 (13)

Symmetry code: (i) −*x*, −*y*+1, −*z*+1.

### Hydrogen-bond geometry (Å, º)

Cg is the centroid of the S1/N1/C2–C4 ring.

**Table:**

*D*—H···*A*	*D*—H	H···*A*	*D*···*A*	*D*—H···*A*
O2—H2···O2^ii^	0.84	1.65	2.470 (3)	165
C3—H3···O1^iii^	0.92	2.37	3.280 (2)	170
C4—H4···*Cg*^iv^	0.91 (3)	2.90 (2)	3.688 (2)	146 (1)

Symmetry codes: (ii) −*x*+1, −*y*, −*z*+1; (iii) *x*−1, *y*+1, *z*; (iv) −*x*, *y*+1/2, −*z*+1/2.
